# Evaluation of Maculopathy in Patients Using Hydroxychloroquine

**DOI:** 10.4274/tjo.galenos.2018.67864

**Published:** 2019-06-27

**Authors:** Adem Uğurlu, Maisa Aslanova, Zafer Cebeci, Nur Kır Mercül

**Affiliations:** 1 Erzincan Binali Yıldırım University, Mengücek Gazi Training and Research Hospital, Department of Ophthalmology, Erzincan, Turkey; 2 İstanbul University İstanbul Faculty of Medicine, Department of Ophthalmology, İstanbul, Turkey

**Keywords:** Hydroxychloroquine, maculopathy, cumulative, dose, fundus autofluorescence

## Abstract

**Objectives:**

To determine length of hydroxychloroquine use and cumulative dose and evaluate the ocular effects by 10-2 central visual field test, microperimetry (MP), color fundus photography, optical coherence tomography (OCT), and fundus autofluorescence (FAF) in hydroxychloroquine users.

**Materials and Methods:**

Patients who used hydroxychloroquine continuously for at least 2 years for various connective tissue diseases were included in the study. A total of 300 eyes of 150 patients aged 19-78 years who were followed due to risk of developing hydroxychloroquine maculopathy in the İstanbul University İstanbul Faculty of Medicine Ophthalmology Department between the years 1995-2017 were evaluated. Best corrected visual acuity (BCVA), biomicroscopic, and fundoscopic examination were performed at all visits. MP, FAF, OCT, fundus photography, and central 10-2 visual field examinations were performed 3 times at 6-month intervals.

**Results:**

The mean age of patients was 48.9±10.8 years; 141 (94%) patients were female and 9 (6%) were male. The mean duration of hydroxychloroquine use was 10.5±6.4 (2-30) years. Fifty-six patients had been using the drug for 5 years or less. The mean cumulative drug dose was 754.7±447.2 (146-1825) g. Mean BCVA was 0.02±0.08 LogMAR at all follow-up visits (p=0.999). Mean MP values at the first, second, and third examinations were 14.07±3.24 dB, 14.18±3.35 dB, and 14.54±2.79 dB, respectively (p>0.05). Mean central macular thickness was 221.9±19.8 μm at initial examination, 221.8±19.9 μm at the second visit, and 221.8±19.8 μm at the final visit (p=0.113). There was a weak negative correlation between age and MP values at all three visits (visit 1: p=0.003, r=-0.170; visit 2: p=0.001, r=-0.185, visit 3: p=0.011, r=-0.146). There was statistically significant relationship between MP values and hydroxychloroquine length of use and cumulative dose (p=0.027 and p=0.049, respectively). Duration of use was not associated with changes in 10/2 visual field (p=0.124). There were significant relationships between alterations in FAF and hydroxychloroquine length of use and cumulative dose (p=0.027 and p=0.049, respectively).

**Conclusion:**

FAF alterations were significantly associated with duration of hydroxychloroquine use and cumulative dose. As objective methods are more reliable, examinations such as FAF can be recommended as auxiliary methods in the follow-up and early detection of toxic maculopathy.

## Introduction

Chloroquine is an antimalarial drug with weak anti-inflammatory and immunomodulatory effects. Because it has fewer side effects and is better tolerated than other immunomodulatory drugs, it has been widely used for about 50 years for the treatment of rheumatoid arthritis (RA), systemic lupus erythematosus (SLE), Sjogren’s syndrome, and other autoimmune diseases.^1^

Toxic retinopathy first appeared in the late 1950s with the introduction of chloroquine.^2,3^ Today, hydroxychloroquine has replaced chloroquine in the treatment of connective tissue diseases due to its lower toxicity.^1^ Although the therapeutic and toxic dose ranges of these two drugs differ, the toxic retinopathy they cause is similar.

Chloroquine and hydroxychloroquine are melanotropic and tend to accumulate in melanin-rich tissues such as the retinal pigment epithelium (RPE) and iris/ciliary body.^4^ Bilateral, irreversible retinal damage in the advanced stages leads to permanent reduction in vision. In some patients, there is no visible lesion in the fundus despite visual field loss. While early changes can resolve upon drug discontinuation, changes in later stages may persist even if the drug is discontinued.^2^ Therefore, early detection of the adverse effects of antimalarial drugs on the retina and immediate drug cessation are important.^2,4^

Many assessment methods are used to aid in the detection of retinal toxicity associated with the use of hydroxychloroquine. The Amsler chart, color vision examination, and central visual field testing are used in routine screening. In recent years, fundus autofluorescence (FAF), optical coherence tomography (OCT), microperimetry (MP), and electrophysiological tests have also been increasingly used in the follow-up of these patients.^3,5,6^

The aim of this study was to determine risk factors and evaluate the effectiveness of diagnostic methods such as color fundus photography, 10-2 central visual field testing, MP, OCT, and FAF in monitoring for the development of maculopathy in patients who have used hydroxychloroquine for at least 2 years.

## Materials and Methods

The eyes of patients who had used hydroxychloroquine continuously for at least 2 years due to RA, SLE, Sjogren’s syndrome, or other connective tissue diseases were included in the study. Those with a previously diagnosed anterior segment or retinal disease, glaucoma, or nystagmus, history of vitreoretinal surgery, media opacity preventing posterior segment imaging, and those who did not attend regular follow-up visits were excluded. All patients were informed in detail about the study and their written informed consent was obtained. The study was carried out in accordance with the Declaration of Helsinki and was approved by the local ethics committee.

Age, sex, systemic comorbidities, history of other ocular pathology, and hydroxychloroquine length of use, daily dose, and cumulative dose were recorded for all patients included in the study. All patients included in the study underwent central 10-2 visual field testing, MP examination, and best corrected visual acuity (BCVA) measurement based on a Snellen chart at each of 3 follow-up examinations conducted at 6-month intervals. BCVA values were converted into LogMAR equivalents. After inducing pupil dilation, fundoscopic examination, fundus photography, OCT, and FAF were performed.

For fundus photography, a Zeiss FF 450 plus (Carl Zeiss Meditec AG, Jena) fundus camera was used to capture 50-degree macular images after pupil dilation. Visual field was assessed by automated static visual field testing using a Humphrey computerized visual field device (Carl Zeiss Meditec Inc., Dublin, CA) after applying refractive correction appropriate for the test distance determined before pupil dilation. Central visual field testing was done using a 10-2 threshold central test program, which scans a 10-degree area at intervals of 2 degrees. If the visual field results showed ≥33% false positive or false negative responses or loss of fixation, they were considered unreliable and the patient was invited back to repeat the test. Loss of more than 5 dB at three or more adjacent points or loss exceeding 10 dB at a single point was accepted as visual field deterioration. OCT was conducted using a Spectralis (Heidelberg Engineering, Heidelberg) device to acquire a horizontal scan with 49 sections passing through the macula. Central macular thickness was measured and the foveal, parafoveal, and perifoveal regions were evaluated for losses in the outer retinal layers, losses in the photoreceptor inner segment/outer segment (IS/OS) band, and RPE irregularity/loss. FAF images were also obtained with the Spectralis (Heidelberg Engineering, Heidelberg) device and were evaluated for anomalies in terms of hypo- and/or hyperautofluorescent points in the parafoveal and perifoveal areas. An OCT/SLO (scanning laser ophthalmoscope) (OTI, Toronto, Canada) device was used for microperimetric examination. During testing, stimulus intensity was changed between 0 dB and 20 dB by increments of 1 dB. A 4-2 strategy was implemented during the test, consisting of a total of 74 Goldmann III stimuli within a circular 20-degree area centered on the fovea. Scans with more than 20% false negative or false positive responses were considered unreliable. Average foveal sensitivity was evaluated in dB. The patients’ data were analyzed to identify any changes that occurred during follow-up. The patients were also compared based on their total duration of use and cumulative dose of hydroxychloroquine.

### Statistical Analysis

SPSS (Statistical Package for the Social Sciences) version 22.0 statistical software package was used for statistical analyses. Continuous variables were expressed as mean ± standard deviation and categorical variables as frequencies and percentages. Between-group comparisons were made using Student’s t-test and analysis of variation (ANOVA) for continuous variables, Friedman test was used to compare variables in repeated measures, Wilcoxon’s signed rank test was used for pairwise comparisons of these variables, and Pearson and Spearman correlation tests were used to assess correlations between variables. A ROC curve analysis was performed to enable prediction of the toxic dose of hydroxychloroquine. The results were evaluated within a confidence interval of 95% based on a significance level of p<0.05.

## Results

The study included 300 eyes of 150 patients, 141 (94%) female and 9 (6%) male, with a mean age of 48.9±10.79 (19-78) years. Forty-six (30.7%) of the patients were over the age of 60. Duration of hydroxychloroquine use ranged from 2 to 30 years, with a mean of 10.51±6.44 years. Only 56 (37.3%) patients had used the drug for was less than 5 years. The daily hydroxychloroquine dose used by the patients was 200 mg/day and the mean cumulative dose was 754.69±447.19 (146-1825) g.

Diagnosis was SLE in 73 patients (48.7%), RA in 41 (27.3%), Sjögren’s syndrome in 23 (15.3%), SLE + Sjögren’s syndrome in 4 (2.7%), scleroderma in 3 (2%), RA + Sjögren’s syndrome in 3 (2%), sarcoidosis in 1 (0.7%), SLE + RA in 1 (0.7%), and Antiphospholipid syndrome in 1 (0.7%). No additional ocular pathology was detected in 83 (55.3%) of the patients, while 58 (38.7%) had dry eye, 7 (4.7%) had cataract, and 2 (1.3%) had amblyopia (Table 1).

Mean BCVA was 0.02±0.08 LogMAR at the start of the study and was unchanged at the second and final follow-up visits (p=0.999). Mean central macular thickness (CMT) was 221.9±19.8 µm at the first visit, 221.8±19.9 µm at the second visit, and 221.8±19.8 µm at the final visit (p=0.113).

Fundus photographs were normal in 85% (n=255) of the patients, while nonspecific (location and character inconsistent with antimalarial drug toxicity) RPE changes in the posterior pole were detected in 26 (8.7%) eyes.

Findings in FAF imaging at the start of the study were normal in 284 (94.7%) eyes, whereas parafoveal and/or perifoveal hyperautofluorescent + hypoautofluorescent spots in the macula were detected in 14 (4.7%) eyes and a bull’s-eye appearance was observed in 2 (0.7%) eyes. The patient exhibiting the bull’s-eye lesion had a history of chronic renal failure + chronic hepatitis B and had rapidly developed bull’s-eye maculopathy within a period of 4 years. There was no significant difference between FAF values at the initial, 6-month, and 12-month examinations (p>0.05). New lesions or the enlargement of existing lesions were not observed. However, there was a significant relationship between FAF anomalies and total length of use and cumulative dose of hyrdoxychloroquine (p=0.027 and p=0.049, respectively).

OCT revealed irregularity of the IS/OS band in 19 eyes (6.3%) at initial examination and in 18 eyes (6%) in the 6- and 12-month examinations. RPE irregularity was detected in 18 eyes (6%) at initial examination and in 17 eyes (5.7%) at 6 and 12 months. Bilateral changes were observed in 2 patients, while unilateral minimal sporadic atypical RPE irregularity was detected in 7 patients and were not attributed to hydroxychloroquine toxicity.

MP values did not vary over the course of follow-up and there was no significant difference between measurements (p=0.533). However, there were statistically significant but weak negative correlations between age and initial, second, and final MP sensitivity values (r=-0.170, p=0.003; r=-0.185, p=0.001; and r=-0.146, p=0.011, respectively). A significant relationship was detected between MP values and duration of hydroxychloroquine use and cumulative dose (p=0.027 and p=0.049, respectively).

During the course of the study, central 10-2 visual field test revealed defects in 20 patients in both eyes or in a single eye, and the test was repeated in patients whose scans were evaluated as having low reliability; hydroxylchloriquine was discontinued in these patients after consulting with the rheumatology or dermatology department they were attending for follow-up. The average age of these patients was 49±10.45 (34-67) years, their mean length of drug use 10.25±7.4 (2-30) years, and their mean cumulative dose was 720.9±472.8 (146-1825) g. Six of these 20 patients also showed a decrease in MP values. After discontinuing the medication, 11 patients showed resolution of the scotoma in final visual field testing. No lesions were detected on OCT, FAF, or fundus photography in these patients, and they were evaluated as premaculopathic.

Taken alone, changes in 10-2 central visual field were not associated with length of hydroxychloroquine use (p=0.124) or cumulative dose (p=0.234).

**Figure 1:**
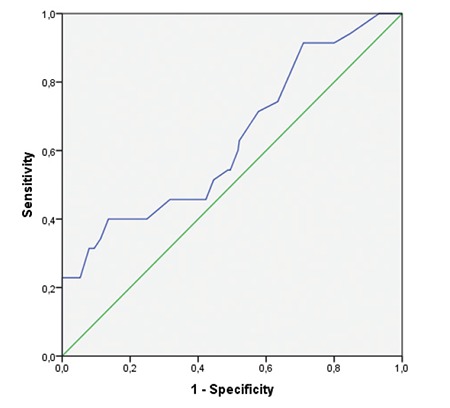
ROC curve analysis Area under the curve: 0.632, SE: 0.053, p=0.11

**Figure 2:**
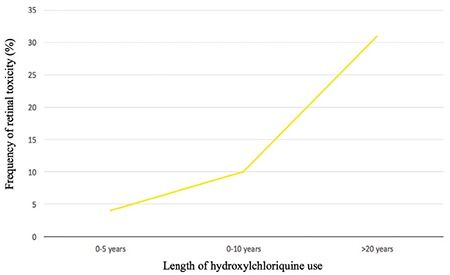
Relationship between length of hydroxychloroquine use and retinal toxicity

Based on patients with defects on FAF and computerized visual field testing and their cumulative drug doses, a ROC curve analysis was done to determine whether a cut-off value for cumulative drug dose could be identified to predict toxicity. A cumulative hydroxychloroquine dose over 425 g indicated higher risk of retinal toxicity with 91% sensitivity and 70% specificity. In addition, the incidence of retinal toxicity increased with duration of hydroxychloroquine use, being approximately 5% in those who used the drug for up to 5 years and 10% in those using for up to 10 years, whereas the incidence increased to 31% among those with over 20 years of use (Figures 1 and 2).

The patients were separated into two groups, those above and below the age of 50 years. When patients with FAF abnormalities and visual field defects were evaluated based on age, there was no significant difference between the two age groups (p=0.313).

## Discussion

FAF examination of the 300 eyes of the 150 patients included in our study showed that total duration of use and cumulative dose of hydroxychloroquine significantly altered the results of this test. According to ROC curve analysis of patients with defects in FAF and automated visual field testing based on amount and duration of drug use, a cumulative hydroxychloroquine dose over 425 grams increased the risk of retinal toxicity with 91% sensitivity and 70% specificity. 

In 2002, the American Academy of Ophthalmology published a recommendation statement reporting that because the cumulative dose poses a more significant risk for hydroxychloroquine toxicity, it should not exceed 460 g.^7,8^ This values is similar to the cumulative dose seen in our study.

Hydroxychloroquine is metabolized and eliminated by the liver and kidneys. Therefore, hydroxychloroquine clearance is reduced in those with liver and kidney disease, increasing the risk of toxicity.^6,9^ This explains the rapid development (within 4 years) of bull’s-eye maculopathy in our patient with history of chronic kidney disease and chronic hepatitis B. This case highlights the need for special attention to the close monitoring of patients at high risk for retinal toxicity caused by antimalarial drugs.

There are not many studies on the evaluation of hydroxychloroquine maculopathy with MP. Martínez-Costa et al.^10^ evaluated 209 patients using hydroxychloroquine with MP and compared the results with those of a control group. They observed no significant difference between the two groups based on age or hydroxychloroquine use. In our study, there was a statistically significant relationship between MP values and the total duration of use and cumulative dose of hydroxychloroquine.

Kellner et al.^11^ compared multifocal electroretinogram (mERG) and FAF imaging to detect early changes in patients who used hydroxychloroquine for over 1 year. Multifocal ERG revealed pericentral, central, and generalized amplitude reduction in all patients with FAF abnormalities and in 4 patients with normal FAF findings. The authors stated that early RPE changes due to antimalarial drug use could be detected accurately with FAF imaging. When mERG and FAF images were compared, it was shown that more retinal anomalies were detected by mERG. The authors recommended discontinuing the drug in patients with retinal anomalies on FAF and mERG.^11^ In another study, Kellner et al.^12^ performed mERG, FAF, and SD-OCT imaging in 8 patients using hydroxychloroquine. FAF revealed pericentral hyperautofluorescent areas and mERG showed pericentral amplitude reduction consistent with these areas.

Bergholz et al.^13^ stated that normal FAF findings could not rule out toxic maculopathy and claimed that OCT and mERG were more sensitive in the early diagnosis of maculopathy.

### Study Limitations

The limitations of our study are that baseline tests could not be performed on patients before they started using hydroxylchloroquine, that all patients were tested at the same time and at 6-month intervals regardless of when they started using the drug, and that more current testing methods like mERG could not be performed.

## Conclusion

Our study demonstrates that care should be taken to ensure that patients at high risk for toxic maculopathy have detailed and regular ophthalmological follow-up. Normal findings in some parameters may be misleading. No matter how meticulously they are applied, methods based on predominantly psychophysical subjective phenomena should not be completely trusted. All kinds of visual field testing and methods based on self-reporting can yield widely varying results over time, even in the same individual. Our study shows that subjective methods should be used in combination with objective methods like FAF in patient follow-up and the early detection of toxic maculopathy. In addition, patients should be more carefully monitored for the development of retinopathy by tracking duration of drug use and approximate cumulative doses, and both patients and rheumatologists should be informed of the risk factors.

## Figures and Tables

**Table 1:**
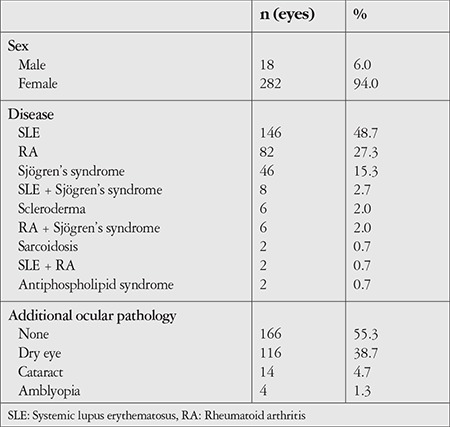
The patients’ sex, additional ocular pathology, and systemic comorbidities
